# Factors Associated With Intent to Leave the Profession for the Allied Health Workforce: A Rapid Review

**DOI:** 10.1177/10775587231204105

**Published:** 2023-10-21

**Authors:** Leonard Roth, Clara Le Saux, Ingrid Gilles, Isabelle Peytremann-Bridevaux

**Affiliations:** 1 Centre for Primary Care and Public Health (Unisanté), University of Lausanne, Switzerland; 2 Lausanne University Hospital, Switzerland

**Keywords:** rapid literature review, allied health workforce, retention issues, health care professionals, intent to leave, intent to stay

## Abstract

Shortages of satisfied and well-trained health care professionals are an urgent threat for health systems worldwide. Although numerous studies have focused on retention issues for nurses and physicians, the situation for the allied health workforce remains understudied. We conducted a rapid review of the literature on allied health workers to investigate the main reasons for leaving their profession. 1,305 original research articles were retrieved from databases MEDLINE, CINAHL, PsycInfo, and Epistemonikos, of which 29 were eligible for data extraction. Reviewed studies featured mainly pharmacists, psychologists, dietitians, physical therapists, emergency medical professionals, and occupational therapists. We categorized 17 typical factors of the intent to leave as organizational, psychological, team and management, and job characteristics. The relative importance of each factor was assessed by measuring its prevalence in the selected literature. By revealing common themes across allied health professions, our work suggests actionable insights to improve retention in these vital services.

## Introduction

Health care professionals (HPs) face specific challenges in their work, such as high workloads and limited resources, which can have a negative impact on their mental and physical health (Bodenheimer & Sinsky, 2014; Lucian Leape Institute, 2013). These challenges are likely to cause high turnover rates in HPs, creating a precarious situation that may lead to a dissatisfied and unprepared workforce (Halter et al., 2017; Shen et al., 2020). This is a major risk for health systems worldwide (Halter et al., 2017; Lucian Leape Institute, 2013; Wakerman et al., 2019). At the same time, health care needs are changing and increasing due to population aging and the rise in non-communicable diseases (Dall et al., 2013; World Health Organization, 2018). With both supply and demand difficulties, there is a growing concern about shortages of HPs (Castro Lopes et al., 2017). Incidentally, the issue is cyclical and systemic as shortages reinforce existing hardships for the health workforce. The COVID-19 pandemic has amplified this phenomenon and showed how little resilient health systems were to crises (American Hospital Association, 2022; Raso et al., 2021). Research can help by investigating factors that compromise the exercise and practice of HPs, as well as the conditions needed for them to thrive (Buchan & Campbell, 2013). In particular, there is a pressing requirement for studies on voluntary departures and the factors that motivate them (Aspden et al., 2021).

Nurses and physicians’ shortages have been clearly recognized and their causes and consequences extensively studied (Halter et al., 2017; OECD, 2008). However, retention issues have also been identified for other health care professions such as physiotherapists, pharmacists, mental health professionals and dietitians (Hawthorne & Anderson, 2009; Hooker et al., 2012; Kakuma et al., 2011; Pretorius et al., 2016). These professions fall into the “allied health workforce” umbrella category, understood here as “trained health professionals, other than nurses and medical practitioners, who are involved in direct patient care or services to the community, or both” (Schoo et al., 2005). Allied health services play a sometimes less visible but none the less vital role for the health system. They have for instance be shown to be more effective and less costly than medical services or drugs in certain cases of chronic disease management (Segal & Robertson, 2004).

A recent survey by AMN Healthcare (2022) of 1,005 health care facilities in the United States indicated that 85% of them were experiencing a shortage of allied health professionals (AHPs). Elsewhere, a report by the Australian National Disability Services (2022) has revealed that difficulties in recruiting and retaining AHPs were driving lengthy wait times for therapy services from 6 months to up to 2 years. The literature suggests that health workforce stability can contribute to reduced costs, improved productivity, and better care outcomes, although most of the research concentrated on nurses (Buchan, 2010). Nevertheless, studies have also pointed out that improving paramedic retention was critical in reducing patient suffering and decreasing mortality (Eubanks, 2022), that high turnover among therapists had both a negative impact on the implementation of evidence-based practices (Woltmann et al., 2008) and could threaten the quality and consistency of mental health services (Babbar et al., 2018), and that continuity of care was desirable for the delivery of effective dietetic services (Hewko et al., 2021).

To the best of our knowledge, very few literature reviews focusing on the retention of HPs have considered multiple allied health professions together. Moreover, all the recent ones concerned the rural and remote setting (Campbell et al., 2012; O’Sullivan & Worley, 2020). Consequently, the evidence for other settings is largely fragmented. Grouping and comparing findings across allied health professions serves a dual purpose: first, consolidating the evidence for each individual profession by recognizing what is common, and second, highlighting meaningful differences by discussing what is not. The primary aim of this review was to examine the factors that contribute to AHPs leaving their profession.

It is often challenging to gather data on individuals who already left their profession. Consequently, we relied on the foundational turnover model by Mobley (1977) to achieve our aim. This work introduced a framework for the employee withdrawal decision process. Crucially, it stated that quit intentions are the most proximal and predictive turnover antecedent. Thus, it opened the door to the study of the intent as a proxy for the decision to leave. This conceptual model was upheld over the years, as demonstrated in a meta-analysis by Griffeth et al. (2000). Grounding our research in this lineage of the turnover theory, we considered interchangeably the last two steps of the withdrawal decision process, that is, quit intentions and the actual decision to quit.

### New Contribution

Retention issues for the allied health workforce have received little attention compared with the research done on nurses and physicians. To the best of our knowledge, this literature review is the first to examine factors associated with intent to leave for multiple allied health professions and without restriction on the work or care setting. By applying a strict methodology, this research highlights general themes that may guide policymakers and managers in improving the working conditions and preventing excessive turnover of AHPs in most common situations.

## Method

We conducted a rapid review of the literature to answer the research question:

Research Question 1 (RQ1): What are the factors associated with intent to leave the profession for the allied health workforce?

Rapid reviews are an efficient tool to support health policymaking and health systems strengthening by synthesizing and presenting evidence in a resource-effective manner (Moher et al., 2015; Tricco et al., 2017). Due to limited resources, the trade-off between exhaustiveness and efficiency was deemed more appropriate for a rapid review than for a systematic review in this case. Rapid reviews usually involve the following steps: (a) setting a research question, (b) establishing eligibility criteria, (c) developing a search strategy, (d) study selection, (e) data extraction, (f) quality assessment, (g) synthesis of findings (Garritty et al., 2021). We used the Preferred Reporting Items for Systematic Reviews and Meta-Analyses statement—a seminal 27-item checklist designed to help authors improve the reporting of systematic reviews and meta-analyses—for the reporting of methods and results where appropriate (Moher et al., 2009).

### Eligibility Criteria

This rapid review was undertaken specifically to inform health workforce planning in Switzerland. Consequently, it included original research articles published in English, German, and French. English is by far the most common language in the academic literature, and German and French were chosen to ensure that all Swiss studies would be captured, as those are the two main Swiss languages. Moreover, since the body of literature concerning Switzerland is very limited, we included all studies taking place in high-income countries. Grouping countries by income, as defined by the World Bank (2022), allows to learn from similarities across country settings. We excluded studies from low- and middle-income countries because these often focus on medical migration (Verma et al., 2016), which implies fundamentally different factors of the intent to leave in comparison with a high-income country such as Switzerland, where health workforce migration is much less of an issue.

Articles published before 2010 were excluded to reflect the fact that working conditions have substantially evolved over the years. Furthermore, we excluded articles centered on COVID-19 because we did not want to focus on the specific conditions that led professionals to leave their profession during the pandemic. The search strategy was constructed around two main concepts: intent to leave the profession and the allied health workforce. We did not impose any restriction on the care setting nor on the design of the original studies.

### Search Strategy

We designed a comprehensive search strategy in consultation with an information specialist. The main database searched was MEDLINE and we complemented the results with searches in specialized databases CINAHL, PsycInfo, and Epistemonikos. The search expressions corresponding to intent to leave included “intention to leave,” ‘turnover “turnover intention” and “reasons for leaving” (see Supplemental Material for all details). We also included expressions such as “intention to stay,” ‘reasons for staying’ and “retention” because a symmetry exists up to a certain point between intent to leave and intent to stay (Cosgrave et al., 2019). These choices were made during group discussions among all authors in the view of ensuring that none of the relevant literature on the topic of interest would be missed.

In the MEDLINE case, the search strategy part corresponding to the allied health workforce included the MeSH term “Allied Health Personnel” as well as an explicit list of all major allied health professions (see Supplemental Material). The search expressions were then adapted to the specialized databases. Based on an initial exploratory search with these terms, a core set of studies was identified and references from their bibliographies screened to further develop the search strategy. The search process and identification of articles were carried out in December 2021.

### Study Selection

First, duplicates across databases were removed. Second, the two reviewers (C.LS. and L.R.) screened each half of all titles and abstracts to select studies that addressed the research question and fulfilled the eligibility criteria. Both reviewers screened the same first hundred articles to calibrate their selection process and minimize bias. Full text was obtained for all studies that passed the initial screening. Then, the two reviewers carried out a further selection based on the full text. Each reviewer read approximately half of all full texts. Studies were selected for data extraction if they explicitly considered factors associated with intent to leave (or its related notions). When studies examined AHPs together with nurses or physicians, we tried separating the results. If it was not feasible, these articles were discarded. Where there was a doubt, the other reviewer checked the selection process until an agreement was reached.

### Data Extraction

The core of each selected article was extracted using a standardized form that contained the following notable elements: study objectives, methodology (qualitative/quantitative/mixed), study design (longitudinal/cross-sectional survey/interviews/focus groups), analytical approach (e.g., descriptive, correlational, thematic analysis), countries involved, participants (profession and number), detailed work setting, socio-demographics, outcome measure, factors associated, and conclusions. We constructed the form so that it fitted both qualitative and quantitative designs. Intent to leave was often studied in conjunction with other outcomes such as job satisfaction. To keep the focus narrow, we only recorded results corresponding to our research question. Data extraction for each article was performed by one reviewer (C.LS. for qualitative and mixed-design studies and L.R. for quantitative studies) and checked by the other.

For this article, we summarized in a tabular form each study’s main characteristics (Table 1). Several studies considered more factors than the ones reported in this table, but we chose to focus on the factors that were found to be associated with the intent to leave/stay and its variations, and discard the ones that were not listed as significant determinants. We did not differentiate in Table 1 between positive and negative associations because of the symmetry in the outcome definition. Finally, the reported analytical approach in this table is each time the most advanced method applied in the study. For quantitative studies, for instance, it goes descriptive < correlation analysis < regression analysis < structural equation modeling. Different analytical approaches imply different ways of identifying associated factors.

**Table 1.** Selected Studies’ Main Characteristics.

Study ID	Methodology	Design	Analytical approach	Countries	Participants	Work setting	Socio-demographics	Outcome measure	Factors associated	Quality assessment
Aspden et al. (2021)	Mixed	Cross-sectional survey and interviews	Regression analysis and thematic analysis	New Zealand	327 pharmacists	Diverse	Women: 60.2%	Decision to leave the profession	Unsupportive professional environment; limited career opportunities; under-utilization of skills and knowledge; lack of recognition; inadequate remuneration	2.54
Boccio et al. (2016)	Quantitative	Cross-sectional survey	Correlational	United States	291 psychologists	School	Women: 80%; mean age: 44.7 years; mean years in the profession: 14.4	Intent to leave current job and intent to leave the profession	Experiencing administrative pressure to behave unethically	2.38
Brown et al. (2010)	Mixed	Interviews	Descriptive and thematic analysis	Australia	31 dietitians	Rural	Women: 90.3%	Retention issues	Work diversity and autonomy; workload; burnout; professional development; limited career opportunities; professional isolation	1.54
Cantu et al. (2022)	Quantitative	Cross-sectional survey	Correlational	United States	340 physical therapists	Diverse	Women: 75.6%	Intent to leave current job in the next 6 months	Ethical workplace environment	2.31
Cash et al. (2018)	Quantitative	Cross-sectional survey	Descriptive	United States	1,248 EMS professionals	Diverse	Women: 39.5%	Decision to leave the profession	Desire for better pay/benefits; further education; dissatisfaction with management; career change; lack of feedback; excessive working hours	2.15
Cash et al. (2019)	Quantitative	Cross-sectional survey	Regression analysis	United States	2,644 EMS professionals	Diverse	Women: 30.2%; mean age: 38 years	Intent to leave current job and intent to leave the profession, both in the next 12 months	Workplace incivility	2.46
Chisholm et al. (2011)	Quantitative	Longitudinal	Survival analysis	Australia	901 allied health professionals	Rural	Women: 86%; mean years in the profession: 3.1	Annual turnover	Profession; age at employment commencement	2.38
Collins (2012)	Quantitative	Cross-sectional survey	Correlational	United States	768 physical therapists	Geriatrics	Mean years in the profession: 4.7	Intent to stay in current job	Relationship with patients/families; pride in work; autonomy; salary and benefits	2.00
Crowe et al. (2018)	Quantitative	Cross-sectional survey	Regression analysis	United States	2,153 EMS professionals	Pre-hospital	Women: 22%	Intent to leave current job and intent to leave the profession, both in the next 12 months	Burnout	2.31
Druwé et al. (2021)	Quantitative	Cross-sectional survey	Regression analysis	Multiple (*n* = 24)	5,099 emergency medical technicians	Diverse	NA	Intent to leave current job	Feels valued and appreciated in the team; team takes time for debriefing; inappropriate medical act	2.23
Ferguson et al. (2011)	Qualitative	Interviews	Thematic analysis	England	26 pharmacists	Diverse	Women: 69.2%	Turnover intention	Lack of recognition; dissatisfaction with organizational management; relationship with line manager	2.00
Fragoso et al. (2016)	Quantitative	Cross-sectional survey	Correlational	United States	102 EMS professionals	Diverse	Women: 44%; mean age: 31.4 years; mean years in the profession: 6.6	Turnover intention	Engagement; burnout	2.00
Gustafsson et al. (2021)	Quantitative	Cross-sectional survey	Correlational	Australia	120 occupational therapists	Academia	Women: 89%	Turnover Intention Scale (TIS-6)	Years in the job	2.23
Hewko et al. (2021)	Qualitative	Interviews	Thematic analysis	Canada	10 dietitians	Public healthcare system	NA	Avoidable turnover	Autonomy; growth opportunities; managerial support; burnout; workload; interprofessional conflict	2.31
Hughes et al. (2011)	Qualitative	Interviews	Content analysis	Australia	28 dietitians	Diverse	Women: 89%; mean age: 34.9 years; mean years in the profession: 9.5	Retention	Variety of work; flexibility; multidisciplinary work; career opportunities; work location; management style; professional isolation	1.77
Leupold et al. (2013)	Quantitative	Cross-sectional survey	Regression analysis	United States	143 pharmacists	Retail	Women: 40%; mean age: 50.6 years; mean years in the profession: 25.2	Intent to leave current organization in the next year and in the next 3 years	Job satisfaction; job embeddedness	2.38
Mak et al. (2013)	Qualitative	Interviews	Thematic analysis	Australia	20 pharmacists	Diverse	Women: 65%	Decision to leave the profession	Lack of recognition; remuneration; excessive working hours; career opportunities; involvement in patient care; dissatisfaction with the work environment	2.46
Porter & Lexen (2022)	Qualitative	Cross-sectional survey	Content analysis	Sweden	1,279 occupational therapists	Diverse	Women: 94%; mean age: 44 years; mean years in the profession: 17	Intent to leave the profession	Stress and high work pressure; remuneration; insufficient leadership; lack of recognition; skills development; working hours	2.08
Rivard, Cash, Woodyard, et al. (2020)	Quantitative	Cross-sectional survey	Descriptive	United States	2,073 EMS professionals	Diverse	Women: 40%; mean age: 36 years	Decision to leave the profession	Further education; Desire for better pay/benefits; stress/burnout	2.23
Rivard, Cash, Chrzan, & Panchal (2020)	Quantitative	Cross-sectional survey	Regression analysis	United States	22,622 EMS professionals	Diverse	Women: 26%; Mean age: 41 years	Intent to leave the profession in the next year and in the next 5 years	Dependence on additional income to make ends meet	2.31
Roncalli & Byrne (2016)	Quantitative	Cross-sectional survey	Correlational	Ireland	77 psychologists	Community mental health	Women: 77%; mean age: 37.8 years; mean years in the profession: 6.7	Turnover intention	Job satisfaction; burnout	2.23
Scanlan et al. (2010)	Quantitative	Cross-sectional survey	Descriptive	Australia	38 occupational therapists	Mental health	Mean years in the profession: 7.7	Turnover intention	Lifestyle reasons; job problems; higher income; career development	1.92
Scanlan & Still (2013)	Quantitative	Cross-sectional survey	Correlational	Australia	34 occupational therapists	Mental health	Women: 79%	Turnover intention	Job satisfaction; disengagement; exhaustion	2.00
Scanlan et al. (2013)	Quantitative	Cross-sectional survey	Regression analysis	Australia	103 occupational therapists	Mental health	Women: 91%	Turnover intention	Job satisfaction; work engagement; disengagement	2.08
Stokes et al. (2010)	Quantitative	Cross-sectional survey	Descriptive	Australia	7,203 psychologists	Diverse	Women: 73%	Reasons for staying in the profession	Flexible working hours; Job satisfaction; income	2.08
Urbonas et al. (2015)	Quantitative	Cross-sectional survey	Structural equation modeling	Lithuania	311 pharmacists	Community health	NA	Michigan Organizational Assessment for turnover intention	Perceived organizational support; organizational commitment	2.54
Watanabe-Galloway et al. (2015)	Qualitative	Focus groups	Thematic analysis	United States	Psychologists	Rural	NA	Retention issues	Salary; disappointing training offer; workload; lack of resources; professional isolation	1.62
Williams et al. (2021)	Quantitative	Cross-sectional survey	Correlational	United States	1,118 dietitians	Diverse	Women: 95%; Mean age: 46.6 years	Intent to leave current job and intent to leave the profession	Work–family enrichment; work–family conflict	2.31
Yanchus et al. (2017)	Quantitative	Cross-sectional survey	Structural equation modeling	United States	2,520 psychologists	Veteran Affairs	Women: 66%	Intent to leave current job	Supervisor support; job satisfaction; emotional exhaustion	2.15

*Note.* EMS = emergency medical services.

### Quality Assessment

We assessed the quality of each selected article with the Quality Appraisal for Diverse Studies (QuADS) tool (Harrison et al., 2021). QuADS was developed to determine the methodological and reporting quality of studies included in systematic reviews when those studies involve heterogeneous study designs as it is the case in our review. QuADS is an improvement over existing tools and was designed principally for health services research (Harrison et al., 2021). It is composed of 13 criteria evaluating elements such as the “theoretical or conceptual underpinning to the research,” the “rationale for choice of data collection tool/s,” the “description of data collection procedure,” or whether “strengths and limitations [were] critically discussed” (see Supplemental Material). The reviewers scored each criterion between 0 and 3 following strict guidelines, where 0 indicates fulfilling none of the requirements for a specific criterion and 3, all of them. Then, those scores were averaged so that each article was assigned an overall quality score between 0 and 3, with 0 indicating low quality according to the QuADS tool and 3, high quality.

### Synthesis of Findings

Selected articles were heterogeneous in terms of methodologies, study designs, analytical approaches, as well as in the ways the outcome and the determinants were measured. Thus, it was not appropriate to perform a meta-analysis, and we undertook a narrative synthesis of the findings—drawing from all the steps described above. Studies designated similar concepts with different wordings, so we operated a correspondence between all factors. This allowed us to highlight main themes that are common across studies. As an additional synthesis step, we categorized these typical factors into four distinct groups.

We designed two synthesis tools to help with the interpretation of the results and visually summarize the extent of research found on the typical factors. First, we noted whether each factor was studied at least once for the principal professions represented in our review (Table 2). Second, we counted the overall number of times each factor was mentioned in the selected literature and displayed those numbers graphically (Figure 2). The corresponding associations had to feature in at least two different articles to be included in these synthesis tools.

**Table 2.** Correspondence Between Typical Factors of the Intent to Leave and Allied Health Professions.

Factor group	Factor	Pharmacist	Psychologist	Dietitian	Physical therapist	EMS professional	Occupational therapist
Job characteristics	Remuneration	X	X		X	X	X
	Work location		X	X			X
	Years in the job						X
Organizational factors	Career opportunities	X		X		X	X
	Workload	X	X	X		X	X
	Professional development		X	X		X	X
	Work environment	X	X		X	X	
	Autonomy			X	X		
	Schedule flexibility		X	X			
	Task diversity			X			
Psychological factors	Burnout		X	X		X	X
	Job satisfaction	X	X				X
	Professional identity	X			X	X	X
	Disengagement						X
Team and management	Leadership	X		X		X	X
	Relationship with manager and peers	X	X	X		X	
	Recognition	X					X

*Note.* A cross indicates that the factor appeared at least once for the corresponding profession in the selected literature. Only professions studied by multiple articles in the rapid review are shown. Typical factors have been classified into four thematic groups. EMS = emergency medical services.

## Results

**Figure 1.**
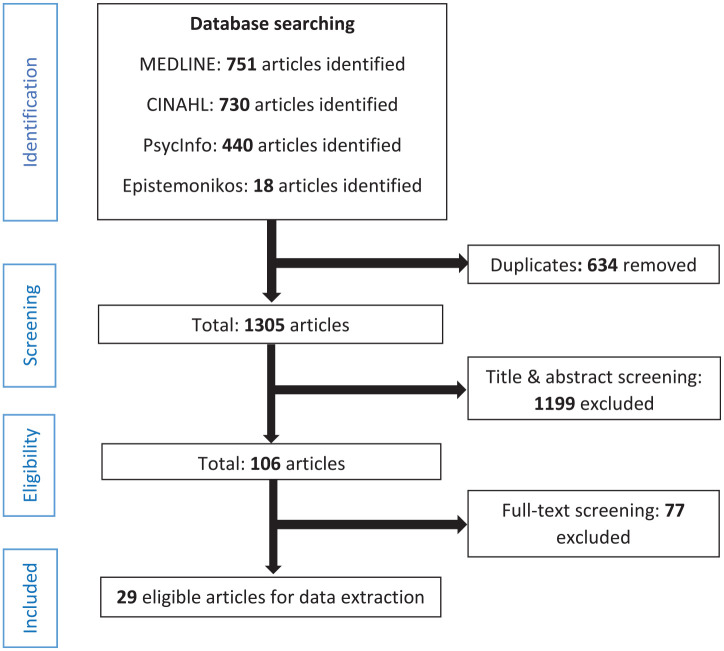
Flow Diagram for the Rapid Review.

After removal of duplicates, 1,305 articles were available for screening by the reviewers. 1,199 articles were excluded based on the title and abstract. Then, a further 77 articles were excluded based on the full text. Out of the 29 articles kept for data extraction, 21 were of quantitative nature, 6 were qualitative, and 2 had a mixed design. All the selected articles analyzed different sets of data and all were in English. Figure 1 provides the flow diagram for the study selection.

### Descriptive Analyses

The countries most represented in our rapid review were the United States (*n* = 13), followed by Australia (*n* = 9). New Zealand, England, Canada, Sweden, Ireland, and Sweden each corresponded to a single study (Table 1). Only one article considered multiple countries together (Druwé et al., 2021). The selected studies focused on seven allied health professions: pharmacists (*n* = 5), psychologists (*n* = 6), dietitians (*n* = 5), physical therapists (*n* = 3), emergency medical services (EMS) professionals (includes emergency medical technicians and paramedics; *n* = 7), occupational therapists (*n* = 6), and speech pathologists (*n* = 1). Those professions were mostly studied on their own, but also a few times in conjunction with other professions (Chisholm et al., 2011; Druwé et al., 2021; Watanabe-Galloway et al., 2015). There were wide variations in the studies’ number of participants, with only articles on psychologists and EMS professionals reporting sample sizes above 1,500 participants (Cash et al., 2019; Crowe et al., 2018; Druwé et al., 2021; Rivard, Cash, Chrzan, & Panchal, 2020; Rivard, Cash, Woodyard, et al., 2020; Stokes et al., 2010; Yanchus et al., 2017).

As indicated in Table 1, several articles omitted socio-demographics information. In the ones were it was mentioned, psychologists (Boccio et al., 2016; Roncalli & Byrne, 2016), dietitians (Brown et al., 2010; Hughes et al., 2011; Williams et al., 2021) and occupational therapists (Gustafsson et al., 2021; Porter & Lexén, 2022; Scanlan et al., 2013) were mostly women (at least 77%), and EMS professionals (Crowe et al., 2018; Rivard, Cash, Chrzan, & Panchal, 2020) were mostly men (at least 74%). For the other occupations, the distribution between women and men was more balanced, although often with a higher proportion of women. The mean participant age varied from 30 years old in a study on EMS professionals (Fragoso et al., 2016) to 50 years old in a study on pharmacists (Leupold et al., 2013). Nine articles reported the average years in the profession, where it varied between 5 years for physical therapists (Collins, 2012) and 25 years for pharmacists (Leupold et al., 2013).

### Factors Identified

**Figure 2.**
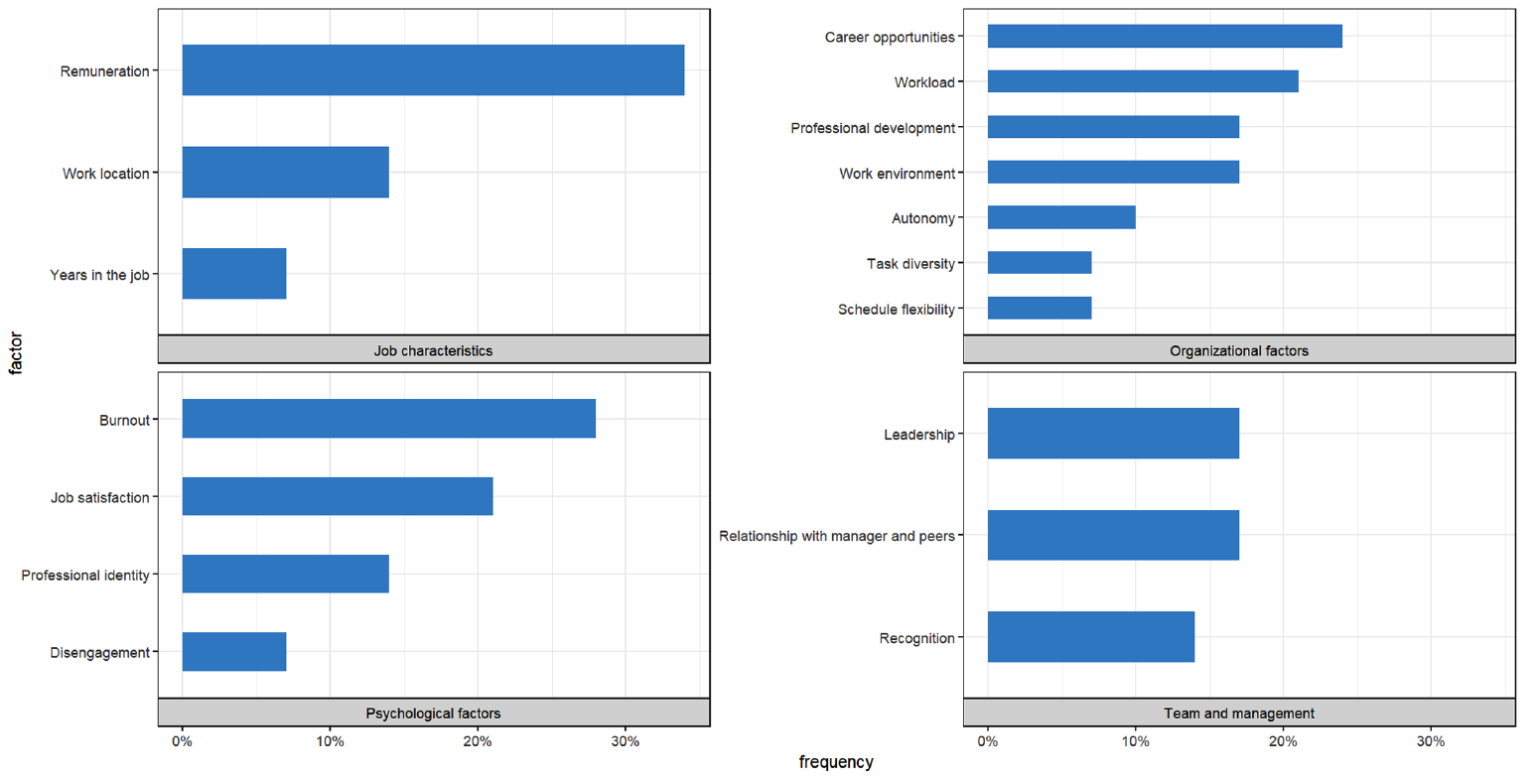
N = 29. The Frequency Represents How Often (in Percentage) Each Typical Factor Was Highlighted in the Selected Literature.

The complete list of factors associated with intent to leave (or its related outcomes) as measured in each article appears in Table 1. They can be categorized as job characteristics, organizational, psychological, and team and management factors. Each category contains between three to seven main factors, presented in Figure 2 in terms of percentage of occurrence in the selected studies.

#### Job Characteristics

Inadequate remuneration was often pointed out as a reason to leave the profession (Aspden et al., 2021; Mak et al., 2013; Porter & Lexén, 2022; Rivard, Cash, Woodyard, et al., 2020; Scanlan et al., 2010), while a competitive salary was highlighted as a reason to stay (Collins, 2012; Stokes et al., 2010; Watanabe-Galloway et al., 2015). A couple times, pay was mixed with benefits (Cash et al., 2018; Rivard, Cash, Woodyard, et al., 2020). Where it was considered separately, salary was more important than benefits (Collins, 2012).

Work location was linked to retention issues when it led to professional isolation (Brown et al., 2010; Hughes et al., 2011; Scanlan et al., 2010; Watanabe-Galloway et al., 2015). When work was conveniently close to home, it had a positive impact on retention (Hughes et al., 2011).

AHPs who were < 30 years old at employment commencement and who spent >5 years in the same position had a higher turnover risk (Chisholm et al., 2011; Gustafsson et al., 2021).

#### Organizational Factors

Limited career opportunities, or a lack of career progression to specialized or higher graded positions, was an important reason for quitting (Aspden et al., 2021; Brown et al., 2010; Mak et al., 2013) and led to avoidable turnover (Hewko et al., 2021; Hughes et al., 2011). Career opportunities elsewhere were also cited as a factor contributing to the decision to leave one’s position (Cash et al., 2018; Scanlan et al., 2010).

High workload was broadly reported as contributing to retention issues (Brown et al., 2010; Cash et al., 2018; Hewko et al., 2021; Mak et al., 2013; Porter & Lexén, 2022; Watanabe-Galloway et al., 2015). Where it was specified, heavy workload corresponded to an excessive number of hours worked (Cash et al., 2018; Mak et al., 2013), or, similarly, to a discrepancy between the tasks at hand and the amount of hours that are expected (Hewko et al., 2021).

Not being able to access professional development was associated with retention issues (Brown et al., 2010; Watanabe-Galloway et al., 2015). Consequently, pursuing further education was a cause underlying turnover (Cash et al., 2018; Porter & Lexén, 2022; Rivard, Cash, Woodyard, et al., 2020).

Several studies mentioned dissatisfaction with the work environment as a reason for leaving the profession (Aspden et al., 2021; Boccio et al., 2016; Cantu et al., 2022; Cash et al., 2019; Mak et al., 2013). Professional environments that led to premature exits were described as unsupportive (Aspden et al., 2021), unethical (Boccio et al., 2016; Cantu et al., 2022), and uncivil (Cash et al., 2019).

A sense of autonomy in the workplace, or control over day-to-day work decisions, helped retain AHPs (Brown et al., 2010; Collins, 2012) and was significantly associated with reduced turnover intention (Hewko et al., 2021).

Although schedule flexibility was rated a positive factor influencing retention among professionals working in private practice (Hughes et al., 2011; Stokes et al., 2010), a lack of flexibility with working hours was reported in other settings (Hughes et al., 2011).

The variety of work, or task diversity, was also a positive determinant of workforce retention (Brown et al., 2010; Hughes et al., 2011).

#### Psychological Factors

Burnout had two main conceptualizations: a tri-partite syndrome comprising the elements of emotional exhaustion, depersonalization, and reduced personal accomplishment (Roncalli & Byrne, 2016); a high degree of physical and psychological fatigue and exhaustion with personal, work-related, and patient-related dimensions (Crowe et al., 2018; Fragoso et al., 2016). A couple of studies focused on the emotional exhaustion aspect (Scanlan & Still, 2013; Yanchus et al., 2017) and Rivard et al. considered burnout intertwined with stress. Burnout was linked to high staff turnover (Brown et al., 2010; Hewko et al., 2021; Rivard, Cash, Woodyard, et al., 2020) and turnover intention (Crowe et al., 2018; Fragoso et al., 2016; Roncalli & Byrne, 2016; Scanlan & Still, 2013; Yanchus et al., 2017).

Job satisfaction is a quite general concept that measures for instance the degree to which a worker has a positive attitude and emotional state regarding the appraisal of the current job situation (Leupold et al., 2013). In the reviewed articles, it was negatively associated with turnover intention (Leupold et al., 2013; Roncalli & Byrne, 2016; Scanlan et al., 2013; Scanlan & Still, 2013; Yanchus et al., 2017) and positively associated with retention (Stokes et al., 2010). However, it had a somewhat different status than the other factors because several articles studied it as an outcome in its own right (Boccio et al., 2016; Ferguson et al., 2011; Rivard, Cash, Chrzan, & Panchal, 2020; Scanlan et al., 2013; Williams et al., 2021).

Professional identity is devised as an umbrella term grouping work engagement (Fragoso et al., 2016; Scanlan et al., 2013), pride in work (Collins, 2012) and organizational commitment (Urbonas et al., 2015). It was a strong predictor of the intent to leave or stay in an organization (Collins, 2012; Fragoso et al., 2016; Scanlan et al., 2013; Urbonas et al., 2015).

Disengagement is closely related to burnout but was sometimes studied independently as the opposite of work engagement (Scanlan et al., 2013; Scanlan & Still, 2013).

#### Team and Management Factors

Dissatisfaction with organizational management and organizational support was a factor influencing the decision to quit (Cash et al., 2018; Ferguson et al., 2011; Porter & Lexén, 2022; Urbonas et al., 2015). Leadership shortfalls included a lack of clear structure (Porter & Lexén, 2022) and a failure to communicate effectively (Ferguson et al., 2011). Hughes et al. (2011) recommended implementing a management restructure aimed at reducing bureaucratic inertia to enhance retention.

Poor relationship with the line manager and peers was an antecedent to turnover (Cash et al., 2018; Druwé et al., 2021; Ferguson et al., 2011; Hewko et al., 2021; Yanchus et al., 2017). The most important aspects of this relationship were supervisory support (Druwé et al., 2021; Ferguson et al., 2011; Hewko et al., 2021; Yanchus et al., 2017) and team feedback (Cash et al., 2018; Druwé et al., 2021).

Several studies directly cited a lack of recognition from management (Ferguson et al., 2011; Mak et al., 2013), colleagues (Porter & Lexén, 2022) and other HPs (Aspden et al., 2021) as a reason for leaving the profession.

Remuneration, career opportunities, workload, professional development, work environment, burnout, professional identity, and leadership were each reported for most of the allied health professions considered in this literature review (at least four out of six, see Table 2) and are thus common factors of the intent to leave/stay. Relationship with manager and peers was also identified as a factor for most professions, although it is mainly relevant for professionals working in teams. Dietitians distinguished themselves from other AHPs by highlighting work diversity and autonomy as an important part of their activity (Brown et al., 2010; Hewko et al., 2021; Hughes et al., 2011). The lack of recognition seemed to be an issue affecting pharmacists in particular (Aspden et al., 2021; Ferguson et al., 2011; Mak et al., 2013). Finally, disengagement was primarily studied on professionals exercising in a mental health setting (Scanlan et al., 2013; Scanlan & Still, 2013). A few other factors were only reported for specific professions and care settings (see Table 1).

### Quality Assessment

The articles’ QuADS scores ranged from 1.5 to 2.5, with most studies obtaining an overall quality score above two (out of three). On the one hand, we did not find any grounds based on these scores to doubt findings from the reviewed articles. On the other hand, several studies failed to mention or insufficiently reported criteria from the quality appraisal, which shows the importance of combining results to attain a higher level of confidence in the findings.

The exact scores assigned to each criterion by the reviewers are available in the Supplemental Material. “Justification for analytic method selected” and “evidence that the research stakeholders have been considered in research design or conduct” had the lowest average scores, indicating that the authors often neglected these elements. In opposition, most articles contained an explicit and detailed “statement of research aim/s” and a “clear description of research setting and target population.”

## Discussion

This rapid review consolidates the evidence from the literature on factors that affect (positively or negatively) AHPs’ work life. By collecting fragmented results and summarizing them into general themes, it provides actionable insights on how to improve the working conditions of AHPs in most common situations. Thus, more than any other factor, adequate remuneration was highlighted as essential to keep AHPs in the workforce. Financial rewards are not however the only significant reason for staying in one’s job and may be complemented with organizational improvements. Our results suggest that organizations wishing to retain their AHPs should give them opportunities for career progression and professional development, or else they may leave to find these opportunities elsewhere. It is also important for AHPs that their working hours are not excessive and that their workload is in line with their working time. Furthermore, promoting professional environments that are ethical and supportive will help prevent AHPs from leaving their job. Where relevant, managerial support is also key. To inspire AHPs, leadership should put effective communication at its center. Finally, our findings acknowledge the fundamental link between psychological factors and retention, thus reiterating the importance of recognizing and preventing burnout early, by for instance providing individual and group supports for AHPs to enhance their mental health and well-being and develop their resilience to work stressors. Differences were also observed across allied health professions, which inform on the positive and negative elements of specific settings. Hence, task diversity and autonomy should be preserved to prevent dieticians from leaving their professions. Besides, a particular focus should be put on improving the recognition toward pharmacists’ activities. Several factors leading to excessive turnover (high workload and burnout for instance) have been amplified during the COVID-19 pandemic. However, this crisis has also shed light on the working conditions of HPs and created an opportunity for change. It is vital for a better post-pandemic recovery to put in place policies and processes that tackle the main underlying determinants of turnover exposed in this paper.

Other recently published literature reviews investigated factors contributing to the recruitment and retention of diverse AHPs, either as the main research question or as secondary outcome, but always restricted to a rural and remote setting (Campbell et al., 2012; Obamiro et al., 2020; O’Sullivan & Worley, 2020; Roots & Li, 2013; Terry et al., 2021). Professional development and career opportunities were identified as key determinants of rural and remote retention issues—for the allied health workforce generally (Campbell et al., 2012; O’Sullivan & Worley, 2020) and for occupational therapists, physiotherapists, and pharmacists specifically (Obamiro et al., 2020; Roots & Li, 2013; Terry et al., 2021). The importance of sufficient workplace supervision was also underlined in the context of reduced density of health care providers, which characterizes this setting (Campbell et al., 2012; O’Sullivan & Worley, 2020; Roots & Li, 2013). Likewise, AHPs in remote areas reported suffering from professional isolation (Campbell et al., 2012), and feeling valued by communities they serve emerged again as a central determinant for the rural pharmacist workforce (Obamiro et al., 2020; Terry et al., 2021). Finally, a factor essential for the rural and remote workforce that appeared only marginally in our rapid review is the training pathway, or how much previous experiences have prepared for the realities of one’s professional activity (Obamiro et al., 2020; O’Sullivan & Worley, 2020; Roots & Li, 2013; Terry et al., 2021).

Findings from literature reviews on the reasons for nurses and physicians to leave their profession share many similarities with what we found for the allied health workforce. For instance, stress and burnout, as well as managerial style, were highlighted as essential determinants of nursing staff turnover in two overviews of reviews (Courvoisier et al., 2023; Halter et al., 2017). When reviewing interventions to improve nurses’ job satisfaction, and thus retention, Niskala et al. (2020) found that strengthening their professional identity was particularly effective. Although professional growth in all its forms is generally recognized as an important determinant of nurses’ turnover intentions (Halter et al., 2017), this is especially true in rural settings (Smith et al., 2019). For physicians, excessive working hours appeared strongly associated with intent to leave (Degen et al., 2015). Thus, the core determinants of a sustainable workforce are common across health professions. A further example of this is a supportive working environment, which is emphasized in a review considering multiple health professions together (Wakerman et al., 2019). Interestingly, Roncalli and Byrne (2016) reported the same risk of burnout in psychologists than in health workers requiring comparatively lower psychological mindedness in the exercise of their professional activity.

Our literature review’s outcome of interest varied from article to article. A few studies reached out to AHPs who already left the profession (Aspden et al., 2021; Cash et al., 2018; Mak et al., 2013; Rivard, Cash, Woodyard, et al., 2020), but the majority evaluated the intentions of AHPs who were still practicing at the time. We based ourselves on the foundational turnover model by Mobley (1977) to use the intent as a surrogate for the decision and thus, to increase the pool of research on which our findings were based. This *modus operandi* has been largely adopted in the turnover literature (Hom et al., 2017). Our results were consistent with Mobley’s theory, since for instance when interviewing pharmacists who left their profession, the most common themes identified by Mak et al. (2013) corresponded exactly to the most common job characteristics and organizational factors highlighted elsewhere in relation to turnover intention (i.e., remuneration, career opportunities, workload, and work environment; see Figure 2). Moreover, Aspden et al. (2021) found when comparing pharmacists who left their profession with pharmacists who were seriously considering leaving that the rationale was very close across the two groups. Notwithstanding, some authors have criticized this framework as insufficient (Halter et al., 2017).

Furthermore, a couple of studies investigated the reasons for *staying* in the profession instead (Collins, 2012; Stokes et al., 2010). Although there exists a significant overlap between intent to leave and intent to stay, they do not measure entirely the same construct (Nancarrow et al., 2014). Finally, some studies assessed intent to leave the current job, some studies assessed intent to leave the profession, and some studies assessed both. Where both were considered, it was always in relation with the same factors (Boccio et al., 2016; Cash et al., 2019; Crowe et al., 2018; Williams et al., 2021). The reported associations were of similar magnitude, indicating a stronger effect on the intent to leave the current job than the profession in all but Crowe et al. (2018). Articles focusing on keeping professionals in specific positions or organizations (Cantu et al., 2022; Collins, 2012; Druwé et al., 2021; Yanchus et al., 2017) may seem less relevant to the general AHPs retention issues but we believe that all findings related to alleviating shortages where they occur can inform strategic interventions elsewhere. The same reasoning applies to the heterogeneity manifest in the outcome’s timescale (from intent to leave in the next 6 months to the next 5 years).

### Limitations

Our results need to be interpreted considering the following limitations. First, the pool of factors examined was not the same in each study, and rarely exhaustive. Consequently, our findings reflect the choice of determinants studied by the authors and not necessarily their absolute importance in predicting the intent to leave, although we postulate that both notions are correlated. Second, the allied health workforce is an umbrella category, which encompasses many professions and various care settings. As such, it was a challenging task to be exhaustive with the search query, and some professions such as speech pathologist or podiatrist were scantly represented in our literature review. Moreover, factors such as leadership style are only meaningful in certain situations and do not apply for instance to independent practitioners. Third, by choosing to exclude studies focusing on the COVID-19 pandemic, we discarded a portion of the recent literature. This decision was taken to ensure that our results were generalizable in the post-pandemic context. It was also motivated by the fact that many health workforce hardships during the pandemic were an extension of already existing problems (French et al., 2022). Finally, a couple of countries were over-represented in our review. This could hamper the generalization to all high-income countries. Nevertheless, we believe that there are more common factors than divergent ones across all kinds of settings. This is supported by the fact that factors such as professional identity were studied for most allied health professions despite their relatively low prevalence in the literature.

### Recommendation for Future Research

All but one of the identified records were based on cross-sectional surveys. Thus, there is a real dearth of longitudinal information on the reasons for staying in or leaving allied health professions. Future studies wishing to fill this gap may gather such information through cohort designs or routinely collected administrative data. The potential advantages of said longitudinal data are multi-fold: investigate how the length of exposure to specific factors influences the intent to leave; explore differences by life/work stages; study the transition from intent to decision; evaluate the effectiveness of interventions designed to keep AHPs in the workforce; bolster the application of state-of-the-art statistical methods for the analysis of professional trajectories, such as Sequence Analysis (Ritschard & Studer, 2018).

## Conclusion

To optimize its performance, a health system needs to enhance patient experience, improve population health, reduce costs, and improve the work life of HPs. In this article, we highlighted retention themes common to several allied health professions. Depending on the resources available, many of these factors constitute levers that can be acted upon. We hope that our findings will inspire initiatives aimed at resolving shortfalls across the allied health workforce, and by doing so, strengthen evidence-based decisions in health human resource planning.

## Supplemental Material

sj-docx-1-mcr-10.1177_10775587231204105 – Supplemental material for Factors Associated With Intent to Leave the Profession for the Allied Health Workforce: A Rapid Review Click here for additional data file. Supplemental material, sj-docx-1-mcr-10.1177_10775587231204105 for Factors Associated With Intent to Leave the Profession for the Allied Health Workforce: A Rapid Review by Leonard Roth, Clara Le Saux, Ingrid Gilles and Isabelle Peytremann-Bridevaux in Medical Care Research and Review
