# Supplementary material for: Factors Associated With Intent to Leave the Profession for the Allied Health Workforce: A Rapid Review
Source: Med Care Res Rev. 2023 Oct 21;81(1):3–18. doi: 10.1177/10775587231204105 (PMC10757398; doi:10.1177/10775587231204105)
Supplement: sj-docx-1-mcr-10.1177_10775587231204105 – Supplemental material for Factors Associated With Intent to Leave the Profession for the Allied Health Workforce: A Rapid Review [file sj-docx-1-mcr-10.1177_10775587231204105.docx]

**Supplemental Material**

For the article titled ‘Factors associated with intent to leave the profession for the allied health workforce: a rapid review’.

**Pages 2 to 6:** Search strategy for databases MEDLINE, CINAHL, PsycInfo and Epistemonikos.

**Pages 7 to 10:** Quality Appraisal for Diverse Studies (QuADS) criteria.

**Page 11:** Quality Appraisal for Diverse Studies (QuADS) scores for all selected studies.

Medline Ovid SP

| **#** | **Search Statement** | **Results** |
| --- | --- | --- |
| 1 | (Personnel Turnover/ or Career Mobility/ or Personnel Loyalty/ or Job Satisfaction/ or ("career development*" or "career mobility" or "career path" or "career paths" or "career pathways" or "career satisfaction*" or "career trajector*" or "career transition*" or demotiv* or "job dissatisfaction" or "job retention" or "job satisfaction" or "job transition*" or "occupational mobility" or "occupational trajector*" or "occupational transition*" or "occupational well-being" or "personnel loyalty" or "professional path" or "professional paths" or "professional pathways" or "professional trajector*" or "recruit and retain" or "recruitment and retention" or "well-being at work" or "work retention" or "work satisfaction*" or "work well-being" or "workplace satisfaction*" or ((intent* or reason*) adj2 (leav* or quit* or stay)) or ((turnover or turn-over) not ((turnover or turn-over) adj3 (bone* or patient*)))).ti,ab,kf.) not (COVID- 19 or SARS-CoV-2 or smoking).ti,ab,hw,kf. not (case stud*.ti. or case report*.ti,pt.) | 139540 |
| 2 | (**Allied Health Personnel/** or Emergency Medical Technicians/ or Nutritionists/ or Occupational Therapists/ or Pharmacists/ or "Physical Therapists"/ or Psychotherapists/ or (dietician* or dietitian* or emergency medical technician* or ergotherapist* or nutritionist* or occupation therapist* or occupational therapist* or paramedic* or speech pathologist* or podiatrist* or pharmacist* or physical therapist* or physiotherapist* or psychologist* or psychotherapist*).ti,kf.) **AND** (Systematic Review/ or meta-analysis/ or meta-analysis as topic/ or (meta analy* or metanaly* or metaanaly* or meta regression).ti. or ((systematic* or evidence*) adj3 (review* or overview*)).ti. or (concept synthesis or conceptual review or critical interpretive synthesis or framework synthesis or integrative review or integrative literature review or literature review or meta-data-analysis or meta-ethnography or (meta adj2 narrative) or meta-study or meta-synthe* or meta-synthe* or mixed method* review or mixed research synthesis or mixed studies review or narrative review or narrative synthesis or realist review or realist synthesis or scoping review or scoping study or qualitative evidence synthesis or qualitative interpretive meta-synthesis or qualitative research synthesis or qualitative systematic review or thematic synthesis or theoretical synthesis or umbrella review).ti.) | 1133 |
| 3 | 1 and 2 | 31 |
| 4 | limit 3 to (abstracts and (english or french or german) and yr="2010 - 2021") | 30 |
| 5 | Emergency Medical Technicians/ or Nutritionists/ or Occupational Therapists/ or Pharmacists/ or "Physical Therapists"/ or Psychotherapists/ or (dietician* or dietitian* or emergency medical technician* or ergotherapist* or nutritionist* or occupation therapist* or occupational therapist* or paramedic* or speech pathologist* or podiatrist* or pharmacist* or physical therapist* or physiotherapist* or psychologist* or psychotherapist*).ti,ab,kf. | 106533 |
| 6 | 5 not 2 | 105522 |

| 7 | 1 and 6 | 1622 |
| --- | --- | --- |
| 8 | limit 7 to (abstracts and (english or french or german) and yr="2010 - 2021") | 751 |
| 9 | 4 or 8 | 781 |

CINAHL EBSCO

((MH "Allied Health Personnel" OR MH "Dietitians" OR MH "Dietitian Attitudes" OR MH "Emergency Medical Technicians" OR MH "Occupational Therapists" OR MH "Occupational Therapist Attitudes" OR MH Pharmacists OR MH "Pharmacist Attitudes" OR MH "Physical Therapists" OR MH "Physical Therapist Attitudes" OR MH "Psychotherapists" OR MH "Psychotherapist Attitudes" OR TI dietician* OR AB dietician* OR TI dietitian* OR AB dietitian* OR TI "emergency medical technician*" OR AB "emergency medical technician*" OR TI ergotherapist* OR AB ergotherapist* OR TI nutritionist* OR AB nutritionist* OR TI "occupation therapist*" OR AB "occupation therapist*" OR TI "occupational therapist*" OR AB "occupational therapist*" OR TI paramedic* OR AB paramedic* OR TI “speech pathologist*” OR AB “speech pathologist*” OR TI podiatrist* OR AB podiatrist* OR TI pharmacist* OR AB pharmacist* OR TI "physical therapist*" OR AB "physical therapist*" OR TI physiotherapist* OR AB physiotherapist* OR TI psychologist* OR AB psychologist* OR TI psychotherapist* OR AB psychotherapist*) **AND** (MH "Systematic Review" OR MH "Meta Analysis" OR TI "meta analy*" OR TI metanaly* OR TI metaanaly* OR TI "meta regression" OR TI ((systematic* OR evidence*) N3 (review* OR overview*)) OR TI "concept synthesis" OR TI "conceptual review" OR TI "critical interpretive synthesis" OR TI "framework synthesis" OR TI "integrative review" OR TI "integrative literature review" OR TI "literature review" OR TI meta-data-analysis OR TI meta-ethnography OR TI (meta N2 narrative) OR TI meta-study OR TI meta-synthe* OR TI meta-synthe* OR TI "mixed method* review" OR TI "mixed research synthesis" OR TI "mixed studies review" OR TI "narrative review" OR TI "narrative synthesis" OR TI "realist review" OR TI "realist synthesis" OR TI "scoping review" OR TI "scoping study" OR TI "qualitative evidence synthesis" OR TI "qualitative interpretive meta-synthesis" OR TI "qualitative research synthesis" OR TI "qualitative systematic review" OR TI "thematic synthesis" OR TI "theoretical synthesis" OR TI "umbrella review")) **OR** ((MH "Dietitians" OR MH "Dietitian Attitudes" OR MH "Emergency Medical Technicians" OR MH "Occupational Therapists" OR MH "Occupational Therapist Attitudes" OR MH Pharmacists OR MH "Pharmacist Attitudes" OR MH "Physical Therapists" OR MH "Physical Therapist Attitudes" OR MH "Psychotherapists" OR MH "Psychotherapist Attitudes" OR TI dietician* OR AB dietician* OR TI dietitian* OR AB dietitian* OR TI "emergency medical technician*" OR AB "emergency medical technician*" OR TI ergotherapist* OR AB ergotherapist* OR TI nutritionist* OR AB nutritionist* OR TI "occupation therapist*" OR AB "occupation therapist*" OR TI "occupational therapist*" OR AB "occupational therapist*" OR TI paramedic* OR AB paramedic* OR TI “speech pathologist*” OR AB “speech pathologist*” OR TI podiatrist* OR AB podiatrist* OR TI pharmacist* OR AB pharmacist* OR TI "physical therapist*" OR AB "physical therapist*" OR TI physiotherapist* OR AB physiotherapist* OR TI psychologist* OR AB psychologist* OR TI psychotherapist* OR AB psychotherapist*)

# AND

(MH "Personnel Turnover" OR MH "Career Mobility" OR MH "Personnel Loyalty" OR MH "Job Satisfaction" OR (TI "career development*" OR AB "career development*" OR TI "career mobility" OR AB "career mobility" OR TI "career path" OR AB "career path" OR TI "career paths" OR AB "career

paths" OR TI "career pathways" OR AB "career pathways" OR TI "career satisfaction*" OR AB "career satisfaction*" OR TI "career trajector*" OR AB "career trajector*" OR OR TI "career transition*" OR AB "career transition*" OR TI demotiv* OR AB demotiv* OR TI "job dissatisfaction" OR AB "job dissatisfaction" OR TI "job retention" OR AB "job retention" OR TI "job satisfaction" OR AB "job satisfaction" OR TI "job transition*" OR AB "job transition*" OR TI "occupational mobility" OR AB "occupational mobility" OR TI "occupational trajector*" OR AB "occupational trajector*" OR TI "occupational transition*" OR AB "occupational transition*" OR TI "occupational well-being" OR AB "occupational well-being" OR TI "personnel loyalty" OR AB "personnel loyalty" OR TI "professional path" OR AB "professional path" OR TI "professional paths" OR AB "professional paths" OR TI "professional pathways" OR AB "professional pathways" OR TI "professional trajector*" OR AB "professional trajector*" OR TI "recruit and retain" OR AB "recruit and retain" OR TI "recruitment and retention" OR AB "recruitment and retention" OR SU "recruitment and retention") OR (TI "well-being at work" OR AB "well-being at work" OR TI "work retention" OR AB "work retention" OR TI "work satisfaction*" OR AB "work satisfaction*" OR TI "work well-being" OR AB "work well-being" OR TI "workplace satisfaction*" OR AB "workplace satisfaction*" OR (TI (intent* OR reason* ) N2 (leav* OR quit* OR stay)) OR (AB (intent* OR reason* ) N2 (leav* OR quit* OR stay)) OR TI ((turnover OR turn- over ) NOT ((turnover OR turn-over) N3 (bone* OR patient*))) OR AB ((turnover OR turn-over ) NOT ((turnover OR turn-over) N3 (bone* OR patient*))))

**NOT** (MH "COVID-19" OR TI "COVID-19" OR AB "COVID-19" OR SU "COVID-19" OR MH "SARS-CoV-2"

OR TI "SARS-CoV-2" OR AB "SARS-CoV-2" OR SU "SARS-CoV-2" OR MH "Smoking Cessation Programs" OR MH "Smoking Cessation" OR TI smoking OR AB smoking OR MH "Case Studies" OR TI "case stud*" OR TI "case report*")

APA PsycINFO

| **#** | **Search Statement** | **Results** |
| --- | --- | --- |
| 1 | (Employee Retention/ or Career Development/ or Occupational Mobility/ or Employee Turnover/ or Job Satisfaction/ or (career development* or career mobility or career path or career paths or career pathways or career satisfaction* or career trajector* or career transition* or demotiv* or job dissatisfaction or job retention or job satisfaction or job transition* or occupational mobility or occupational trajector* or occupational transition* or occupational well-being or personnel loyalty or professional path or professional paths or professional pathways or professional trajector* or "recruit and retain" or "recruitment and retention" or "well-being at work" or work retention or work satisfaction* or work well-being or workplace satisfaction* or ((intent* or reason*) adj2 (leav* or quit* or stay)) or ((turnover or turn-over) not ((turnover or turn-over) adj3 (bone* or patient*)))).ti,ab,id.) not (COVID-19 or SARS-CoV-2 or smoking).ti,ab,hw. not (case stud*.ti. or case report*.ti,sh.) | 59985 |
| 2 | (Allied Health Personnel/ or Paramedics/ or Occupational Therapists/ or Pharmacists/ or Physical Therapists/ or exp Psychotherapists/ or (dietician* or dietitian* or emergency medical technician* or ergotherapist* or nutritionist* or occupation therapist* or occupational therapist* or paramedic* or speech pathologist* or podiatrist* or pharmacist* or physical therapist* or physiotherapist* or psychologist* or psychotherapist*).ti,id.) **AND** (Systematic Review/ or meta-analysis/ or meta- | 267 |

|  | analysis as topic/ or (meta analy* or metanaly* or metaanaly* or meta regression).ti. or ((systematic* or evidence*) adj3 (review* or overview*)).ti. or (concept synthesis or conceptual review or critical interpretive synthesis or framework synthesis or integrative review or integrative literature review or literature review or meta-data- analysis or meta-ethnography or (meta adj2 narrative) or meta-study or meta-synthe* or meta-synthe* or mixed method* review or mixed research synthesis or mixed studies review or narrative review or narrative synthesis or realist review or realist synthesis or scoping review or scoping study or qualitative evidence synthesis or qualitative interpretive meta-synthesis or qualitative research synthesis or qualitative systematic review or thematic synthesis or theoretical synthesis or umbrella review).ti.) |  |
| --- | --- | --- |
| 3 | 1 and 2 | 13 |
| 4 | limit 3 to (abstracts and "0100 journal" and (english or french or german) and yr="2010  - 2021") | 7 |
| 5 | Paramedics/ or Occupational Therapists/ or Pharmacists/ or Physical Therapists/ or exp Psychotherapists/ or (dietician* or dietitian* or emergency medical technician* or ergotherapist* or nutritionist* or occupation therapist* or occupational therapist* or paramedic* or speech pathologist* or podiatrist* or pharmacist* or physical therapist* or physiotherapist* or psychologist* or psychotherapist*).ti,ab,id. | 131890 |
| 6 | 5 not 2 | 131641 |
| 7 | 1 and 6 | 1922 |
| 8 | limit 7 to (abstracts and "0100 journal" and (english or french or german) and yr="2010  - 2021") | 440 |
| 9 | 4 or 8 | 447 |

Epistemonikos

[Title/Abstract]

("allied health personnel" OR dietician* OR dietitian* OR "emergency medical technician" OR "emergency medical technicians" OR ergotherapist* OR nutritionist* OR "occupation therapist" OR "occupation therapists" OR "occupational therapist" OR "occupational therapists" OR paramedic* OR “speech pathologist” OR podiatrist* OR pharmacist* OR "physical therapist" OR "physical therapists" OR physiotherapist* OR psychologist* OR psychotherapist*)

# AND

[Title/Abstract]

("career development" OR "career mobility" OR "career path" OR "career paths" OR "career pathways" OR "career satisfaction" OR "career satisfactions" OR "career trajectory" OR "career trajectories" OR "career transition" OR "career transitions" OR demotiv* OR "intent to leave" OR

"intention to leave" OR "intentions to leave" OR "intention of leaving" OR "intentions of leaving" OR "intent to quit" OR "intention to quit" OR "intent to stay" OR "intention to stay" OR "intentions to stay" OR "job retention" OR "job satisfaction" OR "job satisfactions" OR "occupational well-being" OR "personnel loyalty" OR "professional fulfillment" OR "professional path" OR "professional paths" OR "professional pathways" OR "professional satisfaction" OR "reasons for leaving" OR "reasons to leave" OR "recruit and retain" OR "recruitment and retention" OR "turnover" OR "turn-over" OR "well-being at work" OR "work retention" OR "work satisfaction" OR "work satisfactions" OR "work well-being" OR "workplace satisfaction")

**NOT** ("case study" OR "care report" OR "COVID-19" OR "SARS-CoV-2" OR smoking*)

| **QuADS Criteria** | **0** | **1** | **2** | **3** |
| --- | --- | --- | --- | --- |
| 1. **Theoretical or conceptual underpinning to the research** | No mention at all. | General reference to broad theories or concepts that frame the study.  e.g. key concepts were identified in the introduction section. | Identification of specific theories or concepts that frame the study and how these informed the work undertaken. e.g. key concepts were identified in the introduction section and applied to the study. | Explicit discussion of the theories or concepts that inform the study, with application of the theory or concept evident through the design, materials and outcomes explored.  e.g. key concepts were identified in the introduction section and the application apparent in each  element of the study design. |
| **2. Statement of research aim/s** | No mention at all. | Reference to what the sought to achieve embedded within the report but no explicit aims statement. | Aims statement made but may only appear in the abstract or be lacking detail. | Explicit and detailed statement of aim/s in the main body of report. |
| **3. Clear description of research setting and target population** | No mention at all. | General description of research area but not of the specific research environment e.g. ‘in primary care.’ | Description of research setting is made but is lacking detail e.g. ‘in primary care practices in region [x]’. | Specific description of the research setting and target population of study e.g. ‘nurses and doctors from GP practices in [x] part of [x] city in  [x] country.’ |
| **4. The study design is appropriate to address the stated research aim/s** | No research aim/s stated or the design is entirely unsuitable e.g. a Y/N item survey for a study seeking to undertake exploratory work of lived experiences. . | The study design can only address some aspects of the stated research aim/s e.g. use of focus groups to capture data regarding the frequency and experience of a  disease. | The study design can address the stated research aim/s but there is a more suitable alternative that could have been used or used in addition  e.g. addition of a qualitative or | The study design selected appears to be the most suitable approach to attempt to answer the stated research aim/s. |

|  |  |  | quantitative component could strengthen the design. |  |
| --- | --- | --- | --- | --- |
| **5. Appropriate sampling to address the research aim/s** | No mention of the sampling approach. | Evidence of consideration of the sample required e.g. the sample characteristics are described and appear appropriate to address the research aim/s. | Evidence of consideration of sample required to address the aim. e.g. the sample characteristics are described with reference to the aim/s. | Detailed evidence of consideration of the sample required to address the research aim/s. e.g. sample size calculation or discussion of an iterative sampling process with reference to the research aims or  the case selected for study. |
| **6. Rationale for choice of data collection tool/s** | No mention of rationale for data collection tool used. | Very limited explanation for choice of data collection tool/s. e.g. based on availability of tool. | Basic explanation of rationale for choice of data collection tool/s. e.g. based on use in a prior similar study. | Detailed explanation of rationale for choice of data collection tool/s. e.g. relevance to the study aim/s, co- designed with the target population  or assessments of tool quality. |
| **7. The format and content of data collection tool is appropriate to address the stated research aim/s** | No research aim/s stated and/or data collection tool not detailed. | Structure and/or content of tool/s suitable to address some aspects of the research aim/s or to address the aim/s superficially e.g. single item response that is very general or an open-response item to capture content which requires  probing. | Structure and/or content of tool/s allow for data to be gathered broadly addressing the stated aim/s but could benefit from refinement.  e.g. the framing of survey or interview questions are too broad or focused to one element of the  research aim/s. | Structure and content of tool/s allow for detailed data to be gathered around all relevant issues required to address the stated research aim/s. |
| **8. Description of data collection procedure** | No mention of the data collection procedure. | Basic and brief outline of data  collection procedure e.g. ‘using a questionnaire distributed to staff’. | States each stage of data collection  procedure but with limited detail or states some stages in detail but | Detailed description of each stage  of the data collection procedure, including when, where and how |

|  |  |  | omits others e.g. the recruitment  process is mentioned but lacks important details. | data was gathered such that the procedure could be replicated. |
| --- | --- | --- | --- | --- |
| **9. Recruitment data provided** | No mention of recruitment data. | Minimal and basic recruitment data  e.g. number of people invited who agreed to take part. | Some recruitment data but not a complete account e.g. number of people who were invited and agreed. | Complete data allowing for full picture of recruitment outcomes  e.g. number of people approached, recruited, and who completed with attrition data explained where  relevant. |
| **10. Justification for analytic method selected** | No mention of the rationale for the analytic method chosen. | Very limited justification for choice of analytic method selected. e.g. previous use by the research team. | Basic justification for choice of analytic method selected e.g. method used in prior similar research. | Detailed justification for choice of analytic method selected e.g. relevance to the study aim/s or comment around of the strengths of  the method selected. |
| **11. The method of analysis was appropriate to answer the research aim/s** | No mention at all. | Method of analysis can only address the research aim/s basically or broadly. | Method of analysis can address the research aim/s but there is a more suitable alternative that could have been used or used in addition to offer a stronger analysis. | Method of analysis selected is the most suitable approach to attempt answer the research aim/s in detail  e.g. for qualitative interpretative phenomenological analysis might be considered preferable for experiences vs. content analysis to elicit frequency of occurrence of  events. |
| **12. Evidence that the research stakeholders have been considered in research design or**  **conduct.** | No mention at all. | Consideration of some the research stakeholders e.g. use of pilot study with target sample but no | Evidence of stakeholder input informing the research. e.g. use of pilot study with feedback  influencing the study | Substantial consultation with stakeholders identifiable in planning of study design and in preliminary  work e.g. consultation in the |

|  |  | stakeholder involvement in planning stages of study design. | design/conduct or reference to a project reference group established to guide the research. | conceptualisation of the research, a project advisory group or evidence of stakeholder input informing the  work. |
| --- | --- | --- | --- | --- |
| **13. Strengths and limitations critically discussed** | No mention at all. | Very limited mention of strengths and limitations with omissions of many key issues. e.g. one or two strengths/limitations mentioned with limited detail. | Discussion of some of the key strengths and weaknesses of the study but not complete. e.g. several strengths/limitations explored but  with notable omissions or lack of depth of explanation. | Thorough discussion of strengths and limitations of all aspects of study including design, methods, data collection tools, sample & analytic approach. |

| ID | Theoretical or conceptual underpinning the research | Statement of research aim/s | Clear description of research setting and target population | The study design is appropriate to address the stated research aim/s | Appropriate sampling to address the research aim/s | Rationale for choice of data collection tool/s | The format and content of data collection is appropriate to address the stated research aim/s | Description of data collection procedure | Recruitment data provided | Justification for analytic method selected | The method of analysis was appropriate to answer the research aim/s | Evidence that the research stakeholders have been considered in research design or conduct | Strengths and limitations critically discussed | Total | Study average |
| --- | --- | --- | --- | --- | --- | --- | --- | --- | --- | --- | --- | --- | --- | --- | --- |
| Aspden 2021 | 3 | 3 | 3 | 3 | 2 | 2 | 2 | 3 | 3 | 2 | 2 | 2 | 3 | 33 | 2.54 |
| Boccio 2016 | 3 | 3 | 3 | 2 | 2 | 3 | 3 | 2 | 3 | 1 | 2 | 2 | 2 | 31 | 2.38 |
| Brown 2010 | 1 | 2 | 2 | 2 | 3 | 0 | 2 | 2 | 3 | 1 | 2 | 0 | 0 | 20 | 1.54 |
| Cantu 2021 | 2 | 3 | 3 | 2 | 2 | 3 | 2 | 2 | 3 | 2 | 2 | 2 | 2 | 30 | 2.31 |
| Cash 2018 | 1 | 3 | 3 | 2 | 3 | 2 | 2 | 3 | 3 | 1 | 2 | 1 | 2 | 28 | 2.15 |
| Cash 2019 | 1 | 3 | 3 | 2 | 3 | 2 | 3 | 3 | 3 | 3 | 2 | 1 | 3 | 32 | 2.46 |
| Chisholm 2011 | 2 | 3 | 3 | 3 | 2 | 2 | 3 | 3 | 2 | 2 | 3 | 1 | 2 | 31 | 2.38 |
| Collins 2012 | 2 | 3 | 3 | 2 | 2 | 2 | 2 | 2 | 2 | 1 | 1 | 3 | 1 | 26 | 2.00 |
| Crowe 2018 | 1 | 3 | 3 | 2 | 3 | 2 | 2 | 3 | 3 | 2 | 2 | 3 | 1 | 30 | 2.31 |
| Druwe 2021 | 2 | 3 | 3 | 2 | 2 | 2 | 2 | 2 | 2 | 2 | 3 | 1 | 3 | 29 | 2.23 |
| Ferguson 2011 | 2 | 3 | 3 | 3 | 3 | 1 | 2 | 2 | 2 | 0 | 3 | 0 | 2 | 26 | 2.00 |
| Fragoso 2016 | 3 | 3 | 2 | 2 | 1 | 3 | 2 | 2 | 2 | 2 | 2 | 1 | 1 | 26 | 2.00 |
| Gustasfsson 2021 | 2 | 3 | 3 | 2 | 1 | 3 | 3 | 2 | 3 | 1 | 1 | 2 | 3 | 29 | 2.23 |
| Hewko 2021 | 2 | 3 | 3 | 3 | 1 | 2 | 3 | 3 | 2 | 2 | 3 | 1 | 2 | 30 | 2.31 |
| Hughes 2011 | 2 | 3 | 2 | 3 | 1 | 0 | 3 | 2 | 1 | 0 | 3 | 3 | 0 | 23 | 1.77 |
| Leupold 2013 | 3 | 3 | 3 | 2 | 1 | 3 | 3 | 3 | 3 | 1 | 2 | 1 | 3 | 31 | 2.38 |
| Mak 2012 | 2 | 2 | 3 | 3 | 2 | 2 | 3 | 3 | 2 | 2 | 3 | 3 | 2 | 32 | 2.46 |
| Porter 2021 | 2 | 3 | 2 | 2 | 2 | 1 | 3 | 2 | 3 | 1 | 3 | 0 | 3 | 27 | 2.08 |
| Rivard 2019 | 1 | 3 | 3 | 2 | 3 | 1 | 2 | 3 | 3 | 2 | 2 | 1 | 3 | 29 | 2.23 |
| Rivard 2020 | 2 | 3 | 3 | 2 | 3 | 2 | 2 | 3 | 3 | 2 | 2 | 1 | 2 | 30 | 2.31 |
| Roncalli 2016 | 2 | 2 | 3 | 2 | 2 | 2 | 3 | 3 | 3 | 1 | 2 | 1 | 3 | 29 | 2.23 |
| Scanlan 2010 | 2 | 3 | 2 | 2 | 1 | 2 | 2 | 3 | 3 | 1 | 1 | 1 | 2 | 25 | 1.92 |
| Scanlan & Still  2013 | 3 | 2 | 2 | 2 | 1 | 2 | 3 | 2 | 3 | 2 | 1 | 1 | 2 | 26 | 2.00 |
| Scanlan et al.  2013 | 2 | 3 | 3 | 2 | 2 | 3 | 2 | 2 | 2 | 2 | 2 | 1 | 1 | 27 | 2.08 |
| Stokes 2010 | 2 | 2 | 3 | 3 | 2 | 2 | 2 | 2 | 3 | 1 | 2 | 2 | 1 | 27 | 2.08 |
| Urbanas 2015 | 2 | 3 | 3 | 2 | 2 | 3 | 2 | 2 | 3 | 2 | 3 | 3 | 3 | 33 | 2.54 |
| Watanabe-  Galloway 2015 | 1 | 3 | 3 | 3 | 1 | 0 | 3 | 2 | 1 | 0 | 3 | 0 | 1 | 21 | 1.62 |
| Williams 2021 | 2 | 3 | 3 | 2 | 2 | 3 | 3 | 3 | 3 | 1 | 1 | 3 | 1 | 30 | 2.31 |
| Yanchus 2017 | 3 | 2 | 2 | 2 | 2 | 2 | 2 | 2 | 2 | 2 | 3 | 1 | 3 | 28 | 2.15 |
| **Criteria average** | **2.00** | **2.79** | **2.76** | **2.28** | **1.97** | **1.97** | **2.45** | **2.45** | **2.55** | **1.45** | **2.17** | **1.45** | **1.97** | **28.24** | **2.17** |
